# Preparation of Electrosprayed, Microporous Particle Filled Layers

**DOI:** 10.3390/polym12061352

**Published:** 2020-06-15

**Authors:** Mohanapriya Venkataraman, Kai Yang, Xiaoman Xiong, Jiri Militky, Dana Kremenakova, Guocheng Zhu, Juming Yao, Yan Wang, Guoqing Zhang

**Affiliations:** 1 Department of Material Engineering, Faculty of Textile Engineering, Technical University of Liberec, Studentska 2, 46117 Liberec, Czech Republic; kai.yang@tul.cz (K.Y.); Xiaoman.xiong@tul.cz (X.X.); jiri.militky@tul.cz (J.M.); dana.kremenakova@tul.cz (D.K.); 2 School of Material Science and Engineering, Zhejiang Sci-Tech University, Xiasha Higher Education Zone, Hangzhou 310018, China; zgc100100@hotmail.com (G.Z.); yaoj@zstu.edu.cn (J.Y.); amywang1021@hotmail.com (Y.W.); zgq@zstu.edu.cn (G.Z.)

**Keywords:** PTFE, microporous, electrospraying, contact angle, hydrophobic, electrical resistivity

## Abstract

Polytetrafluoroethylene (PTFE) is a synthetic fluoropolymer known for its excellent hydrophobic properties. In this work, samples from PTFE dispersions with different combinations of water and carbon microparticles were prepared using an electrospraying method. The morphologies and sizes of carbon particles were investigated and the properties of layers including roughness, hydrophobicity and electrical resistivity were investigated. The non-conductive carbon microparticles were selected as a model particle to check the compatibility and electrospraying ability, and it had no effect on the hydrophobic and electrical properties. Carbon microparticles in polymer solution increased the degree of ionization and was found to be beneficial for the shape control of materials. The results showed that PTFE dispersion with the composition of water and carbon microparticles produced fine sphere particles and the layer fabricated with increased roughness. It was also found that the electrical resistivity and hydrophobicity of all the layers comparatively increased. The fabricated microporous layers can be used in various applications like interlining layer in multilayer textile sandwiches.

## 1. Introduction

Polytetrafluoroethylene (PTFE) is a synthetic fluoropolymer with repeated units $\left[{\left(-CF_{2}-\right)}_{n}\right]$, where the inner strongly bonded fluorine atoms make it a high molecular weight compound characterized with semi-crystalline nature, and a strong C-F bond prevents reaction between other chemicals and PTFE [1]. PTFE is known for its excellent hydrophobic, dielectric, mechanical and thermal properties [2]. It is widely used in films, coating materials and fibers in industry domains such as polymeric gears [3], cables [4], implants [5], textiles [6] and so on. Casting PTFE polymer in sphere or PTFE particles via common methods such as molding, emulsion [7], suspension polymerization [8], etc., is very difficult. This is due to the inhomogeneous particle fabrication and a restricted number of processable polymers that cannot be avoided technically. Precipitation [9], spraying drying [10] and supercritical fluid [11] processes are considered to be better options to produce particles. However, it is hard to generate monodisperse particles.

Electrospraying, otherwise called electro-hydrodynamic spraying or electro-hydrodynamic atomization or steady cone-jet mode, is known as a unique technology to produce novel materials including particles, films or coatings [12]. The fundamental mechanism is similar to electrospinning except for the final output. It is a well-established process based on the application of high electrical voltage on the polymer solution of suitable surface tension and viscosity. In this process, the polymer liquid flows out from the capillary nozzle in the applied electric field and then forms a fine jet followed by atomization of polymer liquid into the fine droplets. The particles are collected on a grounded conductive substrate. The degree of ionization in polymer solution affects the shapes and diameters of final polymeric particles during electrospray, which is influenced by the components of the polymer solution. The hydrophobic behavior of the electrospray surface can result from the high surface roughness through reduction of the interfacial energy between solid and liquid [13]. However, a relationship between diameters of PTFE particles and components of PTFE solution will have an effect on sizes, shapes and other properties of PTFE particles produced by electrospraying.

Several research groups have proved that the surface energy of materials was closely related to the surface properties, especially surface roughness [14]. This is a novel way to control material surface wettability. Many theoretical research studies on electrospinning process have been conducted [15,16,17] which showed that distance between nozzle and collector affects morphology, structure, physical and chemical properties of electrospinning fibers and properties based on evaporation rate, deposition time and inconsistency interval. Literature review also revealed that recent research was focused on needle-based electrospraying [18].

The objective of this research was to prepare PTFE microporous layers via electrospraying method [19,20,21] and study its various properties. It would include a study to improve the particles of PTFE by adjusting its solution components and addition of non-conductive carbon microparticles. The relative properties of the prepared PTFE microporous layers including morphology, roughness, hydrophobicity and electrical resistivity were investigated. Addition of carbon microparticles in polymer solution was meant to study the increase in the degrees of ionization and find out if it is beneficial for the compatibility and shape control of materials during electrospinning. In future, it may be possible to add new functionalities via using of activated carbon, conductive carbon or surface coated carbon. The prepared layers can be used for various applications by optimizing the solution and also the spinning parameters.

## 2. Experimental

### 2.1. Materials

**Figure 1:**
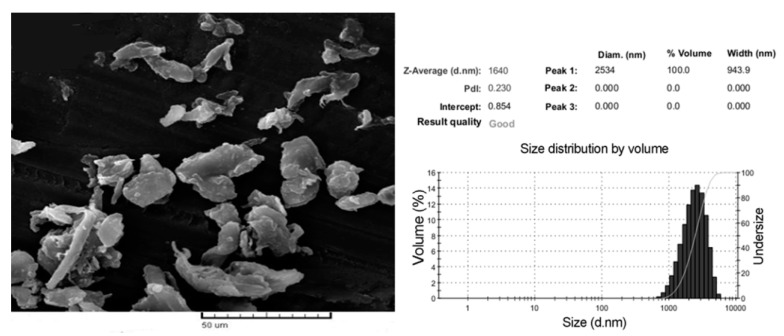
Morphology and size distribution of carbon microparticles.

For this work, Teflon^@^ PTFE 30 containing 60% wt. PTFE particles and 40% wt. water with surfactant (Chemours, Neu Isenburg, Germany) was used. Tetraethylammonium bromide (TEAB) (Sigma Aldrich, Prague, Czech Republic) was used as salt to increase the conductivity and viscosity of solutions in which the interactions among macromolecules are extensive. A polymer network becomes more solid. It leads to a higher spinning performance of a solution [22,23]. Non-conductive carbon microparticles were milled from pitch-based carbon fiber yarn. The milling process was carried out on a Fritsch pulverisette 7 planetary ball mill. The average size of the carbon microparticle was 1640 nm as shown in Figure 1. The PTFE dispersion was obtained after 24 h-stir of Teflon^@^ PTFE 30 mixed with TEAB at room temperature. Non-conductive carbon microparticles were added in PTFE solution before electrospraying to functionalize the final PTFE layers. The details of the PTFE dispersion preparation are shown in Table 1.

### 2.2. Preparation of PTFE Microporous Layers

**Figure 2:**
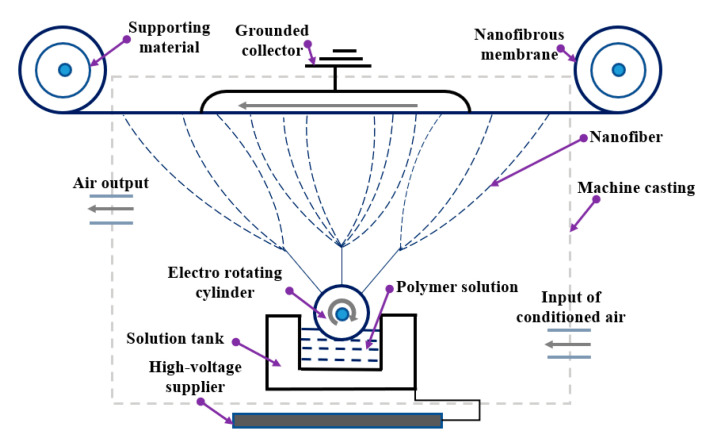
Schematic diagram of the electrospraying method (Nanospider).

The Nanospider instrument (Figure 2) is a needleless electrospinning system that was used to prepare the PTFE layers by electrospraying method. Its parts include a high-voltage supplier, solution tank, rotating roller electrode, grounded collector and support material. During the electrospraying process, the electrostatic force produced between the high-voltage supplier and grounded collector draw the charged polymer solution into forms of particles which will be collected on the surface of support material. The electro-rotating cylinder keeps the electrospraying process ongoing to continuously produce the particles and the even layer of particles is obtained with coordination of the movement of support material.

The PTFE solution was first prepared to be used for electrospraying. As PTFE was insoluble in water, it was introduced in particle form in the water-based dispersion. During the electrospraying process, the PTFE dispersion was drawn to the form layer being collected on the surface of support materials. The particles and their layers are marked as P1, P2, P3 and P4 corresponding to PTFE dispersion S1, S2, S3 and S4. The spinning parameters, including substrate speed (mm/min), applied voltage of high-voltage supplier and grounded voltage (kV), speed of electrode (rev/min), the temperature of air (°C) and the relative humidity (%). The suitable parameters for producing PTFE layers were optimized and selected shown in Table 2. To achieve the optimized conditions, different combinations of solutions were prepared and trialed on Nanospider to set the spinning parameters. The adjusting of process parameters was necessary for the needleless electrospinning system of Nanospider for preparation of layers with tunable porosity.

### 2.3. Characterization of PTFE Microporous Layers

#### 2.3.1. Morphologies

To observe the attachment of the PTFE microporous layer to the surface of support materials, scanning electron microscope (SEM), VEGA TESCAN Inc., Lincoln, NE, USA, was used. The HORIBA laser scattering particle size distribution analyzer LA-920 was used to analyze the size distribution of PTFE particles produced by various PTFE solutions as described in Table 1. The refractive index was set as 1.6. Mean size and S.D. size were calculated as well.

#### 2.3.2. Roughness of PTFE Microporous Layers

The roughness of PTFE layers was tested using the OLS5000 LEXT measuring laser microscope. During the test, every ten different lines of the scanned PTFE layers were recorded and then calculated automatically into the main roughness parameters including arithmetic average height ($R_{a}$), root mean square roughness ($R_{q}$), skewness ($R_{sk}$) and kurtosis ($R_{ku}$), as seen in Figure 2. The $R_{a}$, known as the central line average (CLA), is the most universal parameter for general roughness control given by Equation (1). $R_{q}$ is the parameter describing the standard deviation of the distribution of surface heights and is given by Equation (2). $R_{q}$, $R_{sk}$ and $R_{ku}$ are separately obtained by Equations (3) and (4). $R_{sk}$ is used to measure the symmetry of profile about the mean line and is very sensitive to peak or valley height. The point is that $R_{sk}$ will be positive if the profile has more high peaks or flat valleys and, oppositely, the profile with few peaks gives negative $R_{sk}$ values, contributing to distinguishing the different profiles with the same $R_{a}$ and $R_{q}$. $R_{ku}$ describes the sharpness of the probability density of the profile. If $R_{ku}<3$, the profile will have relatively few high peaks and low valleys and, oppositely, the profile will have relatively more high peaks and low valleys with $R_{ku}>3$.

(1) $$R_{a}=\frac{1}{l_{r}}\int_{0}^{l_{r}}\left|Z\left(x\right)\right|dx$$

(2) $$R_{q}=\sqrt{\frac{1}{l_{r}}}\int_{0}^{l_{r}}Z^{2}\left(x\right)dx$$

(3) $$R_{sk}=\frac{1}{R_{q}^{3}}\left(\frac{1}{l_{r}}\int_{0}^{l_{r}}Z^{3}\left(x\right)dx\right)$$

(4) $$R_{ku}=\frac{1}{R_{q}^{4}}\left(\frac{1}{l_{r}}\int_{0}^{l_{r}}Z^{4}\left(x\right)dx\right)$$

where *x* is the direction of calculation, $l_{r}$ is the reference length along x direction and Z(x) is the height at *x* position.

#### 2.3.3. Contact Angle Analysis

A See System E instrument is a portable computer-based instrument to measure the contact angle with a special purpose software following ISO 27448:2009 test method. Before measuring, the samples were washed for 6 mins in distilled water to remove the salts and were dried at room temperature. Deionized water was dropped onto each PTFE layer from a needle on a microsyringe (5 µL) and a picture of the drop was taken. The contact angles could be calculated by analyzing the shape of the drop, which is displayed in the PC. In total, 10 drops were measured for each sample. Two minutes were allowed from the time of water drop until the measurement of the contact angle.

#### 2.3.4. Electrical Resistance

**Figure 3:**
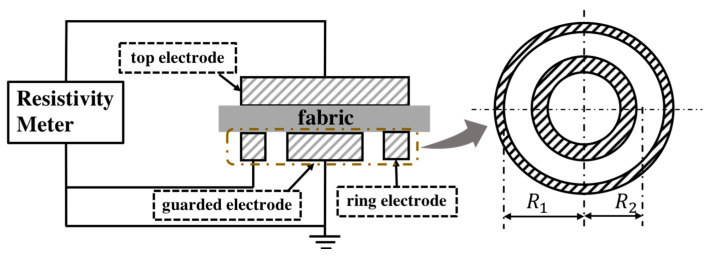
Schematic diagram of an experimental setup to measure electrical resistance.

Electrical resistance measurement was done on a 4339B High resistance meter (Hewlett Packard ohmmeter) measuring device shown in Figure 3. Calibration of the instrument (high resistance meter) is done using a standard plate of known electrical resistivity. The electrical surface and volume resistivity of the samples produced were measured according to the standard ASTM D257-07(2007) at the voltage of 100 V, at a temperature of 22.3 °C, and at a relative humidity (RH) of 40.7%. The measurement results were recorded 60 s after the electrodes were placed on the textile samples. The volume resistivity was measured by applying a voltage potential across opposite sides of the sample and measuring the resultant current through the sample. Volume resistivity *ρ_ν_* (Ω mm) was calculated from the following Equation (5):

(5) $$\rho_{v}=R_{v}\frac{S}{t}$$

where *R_v_*(Ω) is volume resistivity reading, *t* is thickness of the fabric (mm), and *S* is the surface area of the electrodes (mm^2^).

**Figure 4:**
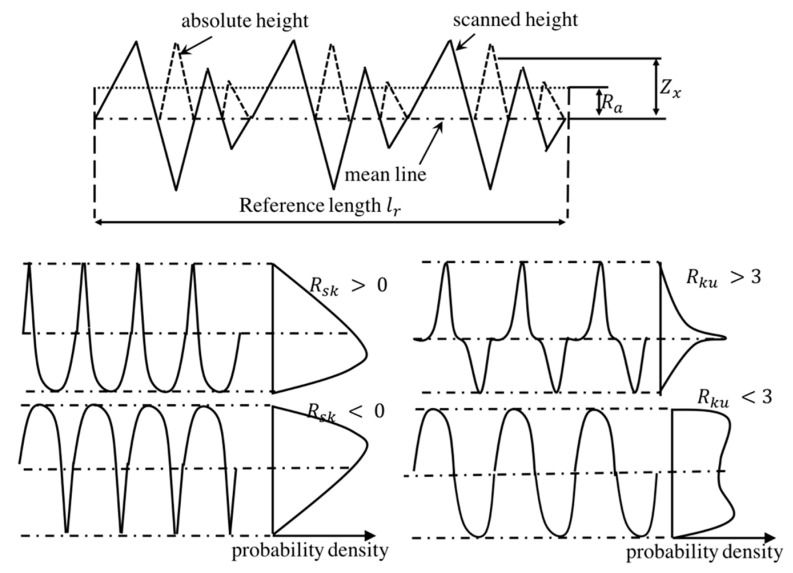
Description of roughness parameters.

Surface resistivity is measured by applying a voltage potential between two electrodes of specified configuration that are in contact with the same side of a material under test (Figure 4). Surface resistivity *ρ_s_* (Ω) was calculated from Equation (6):

(6) $$\rho_{s}=R_{s}\frac{2\pi }{\ln\frac{R_{2}}{R_{1}}}$$

where *R_S_* (Ω) is the surface resistance reading, *R*_1_ is the outer radius of the center electrode (mm), and *R*_2_ is the inner radius of the outer ring electrode (mm), where *R*_1_ = 58 mm and *R*_2_ = 51 mm.

## 3. Results and Discussions

### 3.1. Morphology of Microporous Layers

**Figure 5:**
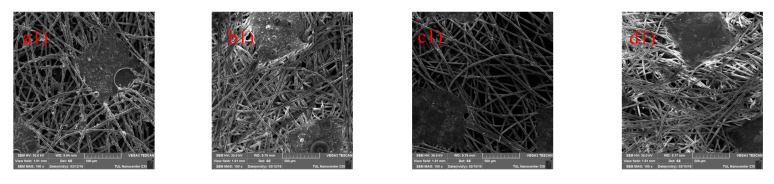

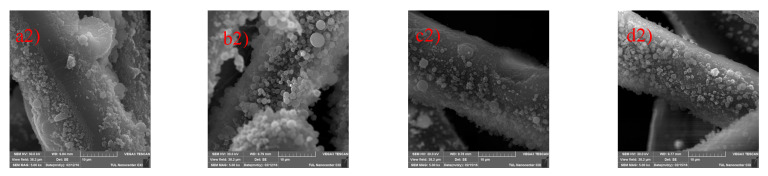
Scanning electron microscope images of PTFE layers (P1: **a1**,**a2**; P2: **b1**,**b2**; P3: **c1**,**c2**; and P4: **d1**,**d2**).

**Figure 6:**
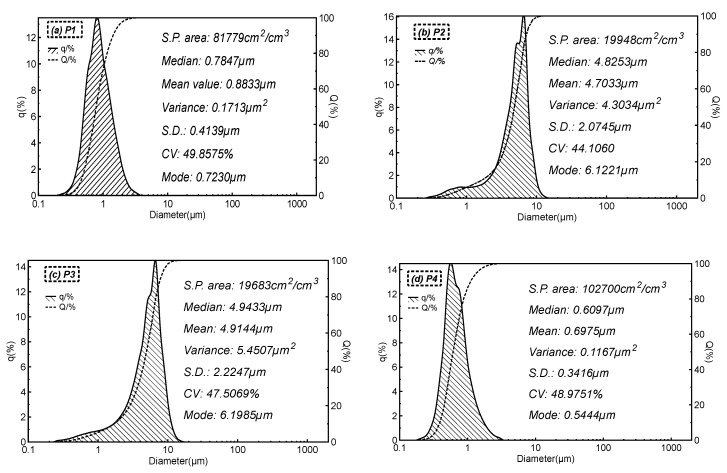
Size distribution of particles (q: density distribution of the particle size; Q: cumulative distribution of the particle size). (**a**) P1; (**b**) P2; (**c**) P3; and (**d**) P4)

The particles on the surface of the substrate were observed in Figure 5 and their size distribution is shown in Figure 6. The layers produced from all combinations of PTFE solution ranges from 0.1–10 µm which proves that the particles are in the range of microns. Figure 5(a2,c2) and Figure 6a,c show the PTFE microporous layers, where the final PTFE product appears in the form of a sphere and has larger sizes when there is more H_2_O in PTFE aqueous solution. In Figure 5(b2), all the layers appear in the form of a sphere as well, which possibly resulted from the carbon microparticles in PTFE polymers. In Figure 5(d2), the particles are almost spherical, but the diameters tend to decrease when compared with Figure 5(b2), which can also be proved by comparing Figure 6b or Figure 6d, suggesting that PTFE dispersion with carbon microparticles and higher water content has the ability to produce fine layers via electrospraying method, which is due to the change of viscosity and surface tension of the solution [24,25,26,27]. Also, the presence of surfactant in the PTFE emulsion attributes to the change in fiber diameter. The formation of finer fibers is facilitated by the presence of surfactant in the solution which increased the conductivity of the solution and decreased the surface tension [28]. The morphology of the microporous membrane depends, on the temperature, solvent, voltage, physical properties of liquid phase, distance of collector, doping agents and the time of solvent evaporation. The fibers tend to form beadlike structures when the concentration of the polymer solution is lower [29,30]. 

### 3.2. Roughness of PTFE Microporous Layers

**Figure 7:**
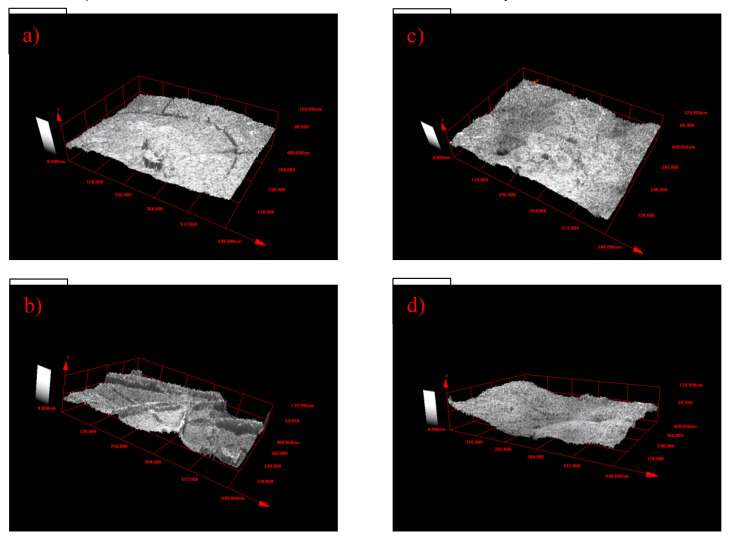
3D morphology of the PTFE microporous layer (**a**) P1; (**b**) P2; (**c**) P3; and (**d**) P4).

Exact distribution of the PTFE layer on the surface of support materials is explained by roughness. Table 3 shows the key parameters of roughness of the PTFE layer, and the 3D morphology of the PTFE microporous layer is shown in Figure 6. PTFE layers P1 and P3, which were separately produced by the S1 and S3 solutions, have the similar surface roughness parameters, shown in Table 3, and the 3D morphologies of the P1 and P3 layers are almost the same as those seen in Figure 7a,c. The $R_{a}$ mean values are much smaller than others, suggesting that the PTFE layer does not have a rough surface compared with the P2 and P4 layers, which may possibly result in a smoother particle shape. However, the P3 layers have $R_{ku}$ values which are smaller than 3, and partial P3 layers have $R_{ku}$ values which are bigger than 3, suggesting that its surface roughness has two kinds of characters. For P2 layers, the $R_{a}$ mean value increases when compared with P1 and P3 layers, which is caused by carbon microparticles in the PTFE polymer, and Figure 7b proves it well. However, all the surface roughness parameters of the P2 layers have no essential difference from the P1 and P3 PTFE layers, and the $R_{sk}$ is negative and all the $R_{ku}$ values are bigger than 3. The $R_{a}$ mean value of the P4 layers increases a lot and reaches 14.617 μm, meaning that it has the largest surface roughness. The P4 layers have a positive $R_{sk}$ mean value and an $R_{ku}$ mean value which is smaller than 3, suggesting that the P4 layer surface is totally different from that of the other three particle layers. Figure 7d shows that the P4 layer tends to be sunken and its surface is even. As described in Section 3.1, with PTFE dispersed in water and with the addition of the right amount of water, the viscosity and the surface tension were adjusted, which in turn led to more evenness of the layers.

### 3.3. Influence on Hydrophobicity

**Figure 8:**
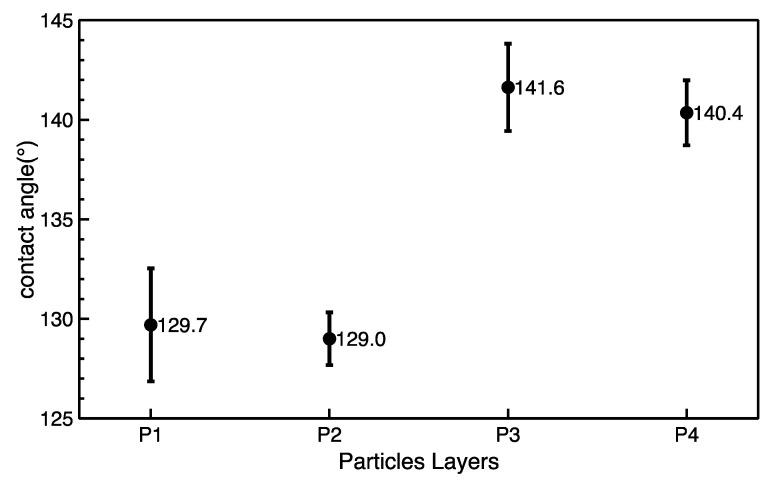
Contact angles after 6 minutes of washing of as-prepared PTFE layers.

**Figure 9:**
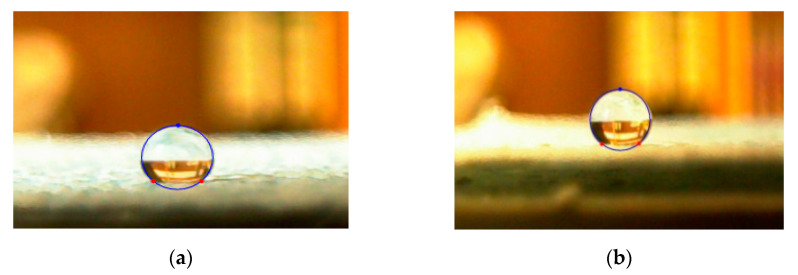
Images of the contact angle measurement: (**a**) 135.3°; (**b**) 142.3°.

The results of the static contact angle measurements are shown in Figure 8 and the digital images of contact angle measurement is shown in Figure 9. The contact angles are over 90°, which proves that all of the particle layers are hydrophobic. The P1 and P2 layers have a close mean value of contact angles, which may be caused by the similarly low $R_{a}$ and $R_{ku}$ values. The P3 and P4 layers have a close higher contact angle. The P4 layer has a contact angle of about 140°. This is because of its high $R_{a}$, given its high contact angle, and $R_{ku}$ values smaller than 3 improve the uniformity of its surface roughness. The surface roughness of the P3 layer explained in Section 3.2 is complex because the P3 layer has $R_{ku}$ values which are bigger than 3 and other parts have $R_{ku}$ values that are smaller than 3. This complex surface roughness of P3 may lead to higher contact angles than those of P4, but the difference is small. As explained in Section 3.2, the adjusted surface tension, evenness and surface roughness of the electrosprayed layer attributes to the hydrophobicity. As seen in Figure 8, samples P3 and P4 showed improved hydrophobicity. Changes in contact angle after 6 minutes of washing (see Figure 8) is probably due to time-dependent partial removal of a water-soluble component other than PTFE which was part of the original PTFE dispersion.

Stability test under washing in standard conditions was conducted for the PTFE layers. It was found that the morphology of layers was the same.

### 3.4. Influence of Electrical Property on Microporous Layers

**Figure 10:**
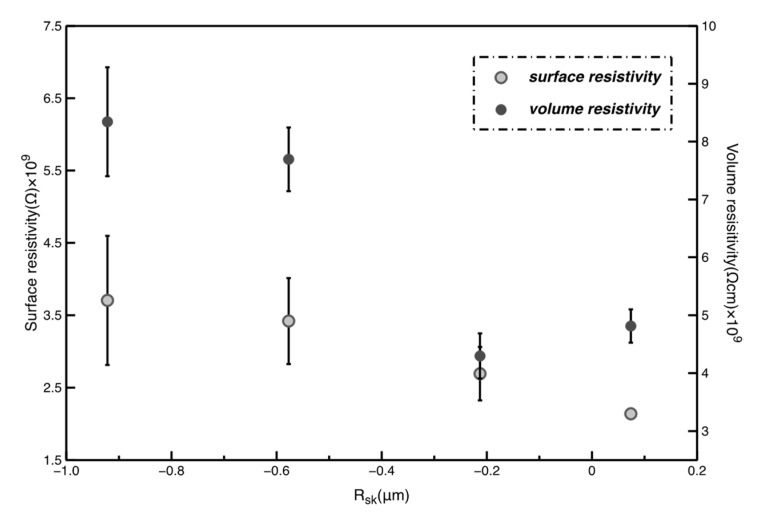
Relationship between resistivity and *R*_sk_.

The thickness and final electrical resistivity values of microporous layers are shown in Table 4. The surface resistivity and volume resistivity of all the layers range in 10^9^ Ω, which means that all of the layers belong to the antistatic body. From the definition of $R_{sk}$, the $R_{sk}$ is the key parameter suggesting the distribution of the continuous layers of materials. The relationship between $R_{sk}$ and resistivity is shown in Figure 10. Surface resistivity slightly decreases with increasing $R_{sk}$, which means that the uniform layers of PTFE increase slightly to improve the movement of electric charges during the resistivity tests. Similarly, the uniform layers of materials also account for volume resistivity, and the volume resistivity values tend to decrease with increasing $R_{sk}$ values. Besides, no clear relationships between contents of carbon microparticles and electrical properties can be found due to the non-conductive property of the carbon microparticles.

## 4. Conclusions

Electrosprayed layers were fabricated from optimized concentration of the solution via electrospraying using the Nanospider instrument. PTFE particles containing carbon microparticles appear in the form of spheres and tend to be smaller with dilution of the PTFE solution. Compared to carbon microparticles added in the PTFE solution, the water content had a greater effect on the surface roughness. An increase of the contact angles of the layers was observed. Surface resistivity and volume resistivity values ranging in 10^9^ Ω were obtained, suggesting that the particle layers were antistatic. Non-conductive carbon microparticles have no significant influence on electrical properties of the layers. However, adding carbon microparticles in polymer solution increased the degrees of ionization and was found to be beneficial for the shape control of materials during electrospraying. By optimizing conditions, more hydrophobic layers can also be achieved.

## Figures and Tables

**Table 1:** Description of PTFE Dispersion (%) Based on different Compositions.

Sample No.	PTFE	Carbon Microparticle	Water & Surfactant
S1	60	-	40
S2	60	0.04	≈40
S3	55	-	45
S4	55	0.04	≈45

**Table 2:** Spinning parameters.

Substrate Speed (mm/min)	Voltage (kV)	Speed of Electrode (rev/min)	In		Tent		Air	
			RH (%)	T (°C)	RH (%)	T (°C)	RH (%)	T (°C)
Static	10/30	5.0	42	22.3	46.8	22.8	49.9	22.4

**Table 3:** Roughness of PTFE microporous layers.

No.	$R_{a}\left(\mu m\right)$	$R_{q}\left(\mu m\right)$	$R_{sk}\left(\mu m\right)$	$R_{ku}\left(\mu m\right)$
P1	6.817 ± 2.700	8.679 ± 3.071	−0.213 ± 0.898	4.038 ± 0.995
P2	7.715 ± 1.212	9.820 ± 1.240	−0.9218 ± 0.233	4.108 ± 0.973
P3	6.690 ± 2.122	8.207 ± 2.479	−0.577 ± 0.285	3.154 ± 1.021
P4	14.617 ± 2.453	17.032 ± 2.495	0.074 ± 0.282	1.989 ± 0.289

**Table 4:** Resistivity values of PTFE layers.

No.	$\mathrm{Surface}\;\mathrm{Resistivity}\left(\Omega \right)\times 10^{9}$	$\mathrm{Volume}\;\mathrm{Resistivity}\left(\Omega cm\right)\times 10^{9}$	t (mm)
P1	2.6924 ± 0.3692	4.2960 ± 0.3901	0.34
P2	3.7044 ± 0.8918	8.3430 ± 0.9417	0.28
P3	3.4196 ± 0.5939	7.6942 ± 0.5509	0.26
P4	2.1392 ± 0.0642	4.8132 ± 0.2878	0.31
